# Supplementary material for: JAK-inhibitors and risk on serious viral infection, venous thromboembolism and cardiac events in patients with rheumatoid arthritis: A protocol for a prevalent new-user cohort study using the Danish nationwide DANBIO register
Source: PLoS One. 2023 Jul 27;18(7):e0288757. doi: 10.1371/journal.pone.0288757 (PMC10374052; doi:10.1371/journal.pone.0288757)
Supplement: S1 Table — (DOCX) [file pone.0288757.s001.docx]

**S1 Table. List of diagnosis code using the International Classification of Diseases 10^th^ revision (ICD-10) for inclusion criteria.**

| Condition | Diagnosis code (ICD-10) |
| --- | --- |
| Seropositive rheumatoid arthritis | M05.X |
| Seronegative rheumatoid arthritis | M06.0 |
| Other specified rheumatoid arthritis | M06.8 |
| Rheumatoid arthritis, unspecified | M06.9 |
|  |  |
