# Supplementary material for: JAK-inhibitors and risk on serious viral infection, venous thromboembolism and cardiac events in patients with rheumatoid arthritis: A protocol for a prevalent new-user cohort study using the Danish nationwide DANBIO register
Source: PLoS One. 2023 Jul 27;18(7):e0288757. doi: 10.1371/journal.pone.0288757 (PMC10374052; doi:10.1371/journal.pone.0288757)
Supplement: S2 Table — (DOCX) [file pone.0288757.s002.docx]

**S2 Table. List of diagnosis code using the International Classification of Diseases 10^th^ revision (ICD-10) and drug therapy using the Anatomical Therapeutic Chemical (ATC) classification codes for antiviral therapy used as exclusion criteria.**

|  | Description | Code |
| --- | --- | --- |
| Diagnosis | Human deficiency virus (HIV) | B20.X - B24.X |
|  | Cancer | C00.X - C96.X (except C44.X) |
|  | Heart transplant | Z94.1 |
|  | Heart valve transplant | Z95.2 - Z95.4 |
|  | Mitral stenosis | I05.X |
|  | Heart valve disorders | I39.0 - I39.4 |
| Antiviral therapy | aciclovir | J05AB01 |
|  | valaciclovir | J05AB11 |
|  | famciclovir | J05AB09 |
|  |  |  |
