# Supplementary material for: JAK-inhibitors and risk on serious viral infection, venous thromboembolism and cardiac events in patients with rheumatoid arthritis: A protocol for a prevalent new-user cohort study using the Danish nationwide DANBIO register
Source: PLoS One. 2023 Jul 27;18(7):e0288757. doi: 10.1371/journal.pone.0288757 (PMC10374052; doi:10.1371/journal.pone.0288757)
Supplement: S3 Table — (DOCX) [file pone.0288757.s003.docx]

**S3 Table. List of drug therapy using the Anatomical Therapeutic Chemical (ATC) classification codes for exposure definition.**

|  | Drug name | ATC code |
| --- | --- | --- |
| TNF-α inhibitors | etanercept | L04AB01 |
|  | infliximab | L04AB02 |
|  | adalimumab | L04AB04 |
|  | certolizumab pegol | L04AB05 |
|  | golimumab | L04AB06 |
| JAK inhibitors | tofacitinib | L04AA29 |
|  | baricitinib | L04AA37 |
|  | upadacitinib | L04AA44 |

Abbreviations: JAK = Janus Kinase, TNF= Tumor Necrosis Factor
