# Supplementary material for: JAK-inhibitors and risk on serious viral infection, venous thromboembolism and cardiac events in patients with rheumatoid arthritis: A protocol for a prevalent new-user cohort study using the Danish nationwide DANBIO register
Source: PLoS One. 2023 Jul 27;18(7):e0288757. doi: 10.1371/journal.pone.0288757 (PMC10374052; doi:10.1371/journal.pone.0288757)
Supplement: S4 Table — (DOCX) [file pone.0288757.s004.docx]

**S4 Table. List of conditions using the International Classification of Diseases 10^th^ revision (ICD-10) for outcome definition.**

|  |  |  |
| --- | --- | --- |
| **Outcome type** | **ICD-10** | **Condition** |
| Major adverse cardiovascular events (MACE) | I21.x - I23.x | Myocardial infarction |
|  | I60.x, I61.x, I62.x, I63.8, I63.9, I64.x | Stroke |
|  | I00 - I09, I11, I13, I20, I25.1-125.9, I25.0, I24, I21-I22, I33, I30 - I31, I40, I50, I26 - I28, I34 - I38, I42 - I49, I51, I10, I12, I15, I60 - I69, I71, I70, I72 - I78, I70, | Cardiovascular death |
| Venous thromboembolism (VTE) | I80.2 | Deep vein thrombosis |
|  | I26.x | Pulmonary embolism |
|  | I80.3, I80.8, I80.9, I81, I74.x, O22.3, O22.5, O22.9, H34.2, H34.8, O87.1, O87.3, O87.9, O88.2, I82.2, I82.3, I82.8, I82.9, M31.1, I51.3, N28.0, K55.0, I67.6, I67.6, I63.4, I63.1, I63.0, I24.0, G08, I66.x, G95.1, I21.9 | Other embolisms |
| Serious viral infection | B25.x | Cytomegaloviral disease |
|  | B27.1 | Cytomegaloviral mononucleosis |
|  | P35.1 | Congenital cytomegalovirus infection |
|  | B27.0 | Mononucleosis due to Epstein-Barr virus |
|  | B02.x | Herpes zoster |
