# Supplementary material for: JAK-inhibitors and risk on serious viral infection, venous thromboembolism and cardiac events in patients with rheumatoid arthritis: A protocol for a prevalent new-user cohort study using the Danish nationwide DANBIO register
Source: PLoS One. 2023 Jul 27;18(7):e0288757. doi: 10.1371/journal.pone.0288757 (PMC10374052; doi:10.1371/journal.pone.0288757)
Supplement: S5 Table — (DOCX) [file pone.0288757.s005.docx]

| Variable | Type of variable | Comments |
| --- | --- | --- |
| Age | continuous |  |
| Sex | Binary:  female male |  |
| Socioeconomic status | categorical |  |
| Disease duration (time since Rheumatoid Arthritis diagnosis) | categorical:  < 1 year 1- 4.9 years 5-10 year >10 years missing |  |
| Smoking status | categorical:  past current never missing |  |
| Pregnancy | binary | At T_0_ looking back up to 365 days |
| Indicators of disease severity: |  |  |
| Health Assessment Questionnaire (HAQ) | categorical:  0 - 0.9 1 - 1.9 2 – 3 missing |  |
| 28-joint disease activity score (DAS28) | categorical:  remission (0–2.6)  low (2.7–3.2) intermediate (3.3–5.0) high (>5.0)  missing |  |
| Rheumatoid factor | categorical:  positive negative missing |  |
| Anti-citrullinated protein (anti-CCP) antibodies | categorical:  positive negative missing |  |
| Patient global disease activity | continuous (visual analogue scale) |  |
| Physician global disease activity | continuous (visual analogue scale) |  |

**S5 Table. List of covariates for Time-Conditional Propensity Score (TCPS).**

T_0_ = study cohort entry date for Janus Kinase (JAK) inhibitors users and the corresponding matching date for Tumor Necrosis Factor (TNF)-α inhibitor users.

**Cont. S5 Table. List of covariates for Time-Conditional Propensity Score (TCPS).**

| Variable | Type of variable | Comments |
| --- | --- | --- |
| Comorbidities: |  |  |
| Viral infection (as per outcome defintion) | binary | Record between  base cohort entry (incl. base cohort entry date) and  study cohort entry date. |
| Thrombosis (as per outcome definition) | binary | Record between  base cohort entry (including base cohort entry date) and  study cohort entry date. |
| MACE (as per outcome definition) | binary | Record between  base cohort entry (including base cohort entry date) and  study cohort entry date. |
| Other cardiac disorders | binary |  |
| Cerebrovascular diseases | binary |  |
| Peripheral vascular diseases | binary |  |
| Hypertension | binary |  |
| Fractures | binary | At T_0_ looking back up to 180 days |
| Major surgery | binary | At T_0_ looking back up to 365 days |
| Psoriasis | binary |  |
| Other rheumatic conditions | binary |  |
| Transplanted organ | binary |  |
| Asthma | binary |  |
| Chronic obstructive pulmonary disease (COPD) | binary |  |
| Diabetes | binary |  |
| Alzheimer’s disease | binary |  |
| Epilepsy | binary |  |
| Dementia | binary |  |
| Depression | binary |  |
| Parkinson’s disease | binary |  |
| Osteoporosis | binary |  |
| Prolonged immobility | binary |  |
| Renal failure | binary |  |
| Chronic kidney disease | binary |  |
| Glomerular disorders | binary |  |
| Cystic kidney disease | binary |  |
| Liver diseases | binary |  |
| Ulcerative colitis | binary |  |
| Other noninfective gastroenteritis and colitis | binary |  |
| Chron disease | binary |  |
| Thrombophilia | binary |  |

Abbreviations: MACE major cardiovascular event. T_0_ = study cohort entry date for Janus Kinase (JAK) inhibitors users and the corresponding matching date for Tumor Necrosis Factor (TNF)-α inhibitor users.

**Cont. S5 Table. List of covariates for Time-Conditional Propensity Score (TCPS).**

Abbreviations: ACE angiotensin-converting enzyme; TNF-α tumor necrosis factor alpha; NSAIDs non-steroidal anti-inflammatory drugs. T_0_ = study cohort entry date for Janus Kinase (JAK) inhibitors users and the corresponding matching date for Tumor Necrosis Factor (TNF)-α inhibitor users.

| Variable | Type of variable | Comments |
| --- | --- | --- |
| Medication: |  |  |
| Previous immunization for Herpes Zoster | binary | In the prior 5 years to T_0_ |
| Antiviral agents (except acyclovir, valaciclovir and famciclovir) | binary | In the prior 365 days to T_0_ |
| Other anti-infective drugs |  | In the prior 365 days to T_0_ |
| Glucocorticoids use (cumulative dose in the past year) | continuous | In the prior 365 days to T_0_ |
| Conventional synthetic disease-modifying antirheumatic drug (csDMARD) medication | binary | At T_0_ |
| Number of previous biologics (non- TNF-α inhibitor) | 1  >1 | Ever prior to study cohort entry |
| Other TNF-α inhibitor previous to study cohort entry date | binary | Ever prior to study cohort entry |
| NSAIDs | binary |  |
| Antidepressants | binary |  |
| Anti-epileptics | binary |  |
| Antipsychotics | binary |  |
| Anxiolytics | binary |  |
| Anti-hypertensives | binary |  |
| Diuretics | binary |  |
| Beta blockers | binary |  |
| Calcium channel blockers | binary |  |
| ACE inhibitors | binary |  |
| Angiotensin II blockers | binary |  |
| Statins | binary |  |
| Lipid lower agents | binary |  |
| Hormone replacement therapy | binary |  |
| Oral contraceptive | binary |  |
| Non-oral contraceptive | binary |  |
| Insulin | binary |  |
| Non-insulin oral anti-diabetic drugs (NIADs) | binary |  |
| anti-thrombotic agents (non-acetylsalicylic acid based) | binary |  |
| Acetylsalicylic acid | binary |  |
| Opioids | binary |  |
