# Supplementary material for: JAK-inhibitors and risk on serious viral infection, venous thromboembolism and cardiac events in patients with rheumatoid arthritis: A protocol for a prevalent new-user cohort study using the Danish nationwide DANBIO register
Source: PLoS One. 2023 Jul 27;18(7):e0288757. doi: 10.1371/journal.pone.0288757 (PMC10374052; doi:10.1371/journal.pone.0288757)
Supplement: S6 Table — (DOCX) [file pone.0288757.s006.docx]

**S6 Table. List of Anatomical Therapeutic Chemical (ATC) classification codes for definition of comedication.**

| Drug | ATC code | Comments |
| --- | --- | --- |
| Glucocorticoids for systemic use | H02AB (except H02AB06) | baseline use up to 90 days before T_0_ (include dose at baseline) |
| Prednisolone | H02AB06 | baseline use up to 90 days before T_0_ (include dose at baseline) |
| Anti-inflammatory/antirheumatic agents in combination with corticosteroids | M01BA | baseline use up to 90 days before T_0_ (include dose at baseline) |
| Methotrexate | L04AX03 | ever before T_0_ (include dose at baseline) |
| Etanercept | L04AB01 | ever before T_0_ |
| Infliximab | L04AB02 | ever before T_0_ |
| Adalimumab | L04AB04 | ever before T_0_ |
| Certolizumab pegol | L04AB05 | ever before T_0_ |
| Golimumab | L04AB06 | ever before T_0_ |
| Rituximab | L01XC02 | ever before T_0_ |
| Abatacept | L04AA24 | ever before T_0_ |
| Tocilizumab | L04AC07 | ever before T_0_ |
| Sarilumab | L04AC14 | ever before T_0_ |
| Anakinra | L04AC03 | ever before T_0_ |
| Hormonal contraceptives for systemic use | G03A (all codes) |  |
| Androgens | G03B (all codes) |  |
| Estrogens | G03C (all codes) |  |
| Progestogens | G03D (all codes) |  |
| Androgens and Female sex hormones in combination | G03E (all codes) |  |
| Progestogens and estrogens in combination | G03F (all codes) |  |
| Gonadotropins and other ovulation stimulants | G03G (all codes) |  |
| Antiandrogens | G03H (all codes) |  |
| Other sex hormones and modulators of the genital system | G03X (all codes) |  |
| Butylpyrazolidines | M01AA (all codes) |  |
| Acetic acid derivatives and related substances | M01AB (all codes) |  |
| Oxicams | M01AC (all codes) |  |
| Propionic acid derivatives | M01AE (all codes) |  |
| Fenamates | M01AG (all codes) |  |
| Coxibs | M01AH (all codes) |  |
| Other antiinflammatory and antirheumatic agents, non-steroids | M01AX (all codes) |  |
| Antidepressants | N06A (all codes) |  |

T_0_ = study cohort entry date for Janus Kinase (JAK) inhibitors users and the corresponding matching date for Tumor Necrosis Factor (TNF)-α inhibitor users

**S6 Table. List of Anatomical Therapeutic Chemical (ATC) classification codes for definition of comedication.**

| Drug | ATC code | Comments |
| --- | --- | --- |
| Vitamin K antagonists | B01AA (all codes) |  |
| Heparin group | B01AB (all codes) |  |
| Platelet aggregation inhibitors excl. heparin | B01AC (all codes) |  |
| Enzymes | B01AD (all codes) |  |
| Direct thrombin inhibitors | B01AE (al codes) |  |
| Direct factor Xa inhibitors | B01AF (all codes) |  |
| Other antithrombotic agents | B01AX (all codes) |  |
| Antiviral for systemic use | J05 (all codes) | baseline use up to 365 days before T_0_ (include dose at baseline) |
| Antibacterials for systemic use | J01 (all codes) | baseline use up to 365 days before T_0_ (include dose at baseline) |
| Antimycotics for systemic use | J02 (all codes) | baseline use up to 365 days before T_0_ (include dose at baseline) |
| Antimycobacterials | J04 (all codes) | baseline use up to 365 days before T_0_ (include dose at baseline) |

T_0_ = study cohort entry date for Janus Kinase (JAK) inhibitors users and the corresponding matching date for Tumor Necrosis Factor (TNF)-α inhibitor users
