# Supplementary material for: JAK-inhibitors and risk on serious viral infection, venous thromboembolism and cardiac events in patients with rheumatoid arthritis: A protocol for a prevalent new-user cohort study using the Danish nationwide DANBIO register
Source: PLoS One. 2023 Jul 27;18(7):e0288757. doi: 10.1371/journal.pone.0288757 (PMC10374052; doi:10.1371/journal.pone.0288757)
Supplement: S7 Table — (DOCX) [file pone.0288757.s007.docx]

**S7 Table. List of Anatomical Therapeutic Chemical (ATC) classification codes for definition of Herpes Zoster (HZ) immunization.**

| Vaccine name | ATC code |
| --- | --- |
| varicella/zoster immunoglobulin | J06BB03 |
| zoster, live attenuated | J07BK02 |
| zoster, purified antigen | J07BK03 |
