# Supplementary material for: JAK-inhibitors and risk on serious viral infection, venous thromboembolism and cardiac events in patients with rheumatoid arthritis: A protocol for a prevalent new-user cohort study using the Danish nationwide DANBIO register
Source: PLoS One. 2023 Jul 27;18(7):e0288757. doi: 10.1371/journal.pone.0288757 (PMC10374052; doi:10.1371/journal.pone.0288757)
Supplement: Attachment — Submitted filename: Response to reviewers.docx [file pone.0288757.s008.docx]

*Cover Letter*

JAK-inhibitors and risk on serious viral infection, venous thromboembolism and cardiac events in patients with rheumatoid arthritis: A protocol for a prevalent new-user cohort study using the Danish nationwide DANBIO register

To: Dr Ryu Watanabe, M.D., Ph.D.

ETH Zürich

Maria Luisa Faquetti, PhD Candidate

maria.faquetti@pharma.ethz.ch

Prof Dr Andrea Burden

andrea.burden@pharma.ethz.ch

Academic Editor

Dr Emily Chenette

Editor-in-Chief

PLOS ONE

**June 2023**

Dear Dr Watanabe and Dear Dr Chenette

On behalf of every co-author, we would like to thank the reviewers for their feedback. We appreciate the improvement on the manuscript after taking the comments into consideration, and we are happy to submit the revised documents. We here provide the rebuttal letter with a point-by-point response to the comments raised by the academic editor and the reviewers. Additionally, we attach the revised manuscript as both a marked copy (Revised Manuscript with Track Changes) and an unmarked copy (Manuscript). The answers on the point-by-point response refer to page and line numbers on the ‘Revised Manuscript with Track Changes’ document.

Finally, as requested, we have updated the author’s potential conflicts of interest with the following statement: “*RC has received a consultant fee from Galapagos and is employed by IQVIA outside of the present work. LD has received research grant (paid to her institution) from BMS outside the current manuscript. LD is a member of the steering committee of the Danish Rheumatology Quality Registry (DANBIO, DRQ), which receives public funding from the hospital owners and funding from pharmaceutical companies. MLF, EVY, AMB have declared that no competing interests exist. This does not alter our adherence to PLOS ONE policies on sharing data and materials*.”

Kind regards,

**Corresponding author:**

Andrea Burden, Ph.D.

Assistant Professor of Pharmacoepidemiology
Institute of Pharmaceutical Sciences
ETH Zurich, HCI H407
Vladimir-Prelog-Weg 4 
CH-8093 Zurich

andrea.burden@pharma.ethz.ch

Maria Luisa Faquetti,

PhD Candidate

Institute of Pharmaceutical Sciences
ETH Zurich, HCI J474
Vladimir-Prelog-Weg 4 
CH-8093 Zurich

maria.faquetti@pharma.ethz.ch

*Point-by-point response*

# Journal requirements:

## Journal requirement #1

Please ensure that your manuscript meets PLOS ONE's style requirements, including those for file naming. The PLOS ONE style templates can be found at

[https://journals.plos.org/plosone/s/file?id=wjVg/PLOSOne_formatting_sample_main_body.pdf](https://eur03.safelinks.protection.outlook.com/?url=https%3A%2F%2Fjournals.plos.org%2Fplosone%2Fs%2Ffile%3Fid%3DwjVg%2FPLOSOne_formatting_sample_main_body.pdf&data=05|01|enriqueta.vallejo@unibe.ch|a40eb79e005345a92a0208db669c5a96|d400387a212f43eaac7f77aa12d7977e|1|0|638216594270013927|Unknown|TWFpbGZsb3d8eyJWIjoiMC4wLjAwMDAiLCJQIjoiV2luMzIiLCJBTiI6Ik1haWwiLCJXVCI6Mn0%3D|3000|||&sdata=2zSvzcyNmu0ImlDvcAqYLhgG9Cvls%2BSuzG59yFISuhM%3D&reserved=0) and

[https://journals.plos.org/plosone/s/file?id=ba62/PLOSOne_formatting_sample_title_authors_affiliations.pdf](https://eur03.safelinks.protection.outlook.com/?url=https%3A%2F%2Fjournals.plos.org%2Fplosone%2Fs%2Ffile%3Fid%3Dba62%2FPLOSOne_formatting_sample_title_authors_affiliations.pdf&data=05|01|enriqueta.vallejo@unibe.ch|a40eb79e005345a92a0208db669c5a96|d400387a212f43eaac7f77aa12d7977e|1|0|638216594270013927|Unknown|TWFpbGZsb3d8eyJWIjoiMC4wLjAwMDAiLCJQIjoiV2luMzIiLCJBTiI6Ik1haWwiLCJXVCI6Mn0%3D|3000|||&sdata=TXAXAab1olzyD4QJF0gLkoLcdA%2B159un3t5bP%2Bq7%2BjQ%3D&reserved=0)

### Answer

Upon double checking guidelines, we have amended the title, which is currently written in sentence case and have not used italics or bold type. Moreover, we added “Danish” to improve understanding of the data source.

Page 1, lines 1 - 4 (“Title”)

“JAK-inhibitors and risk on serious viral infection, venous thromboembolism and cardiac events in patients with rheumatoid arthritis: A protocol for a prevalent new-user cohort study using the Danish nationwide DANBIO register”.

## Journal requirement #2

Thank you for stating the following in the Competing Interests section:

“I have read the journal's policy, and the authors of this manuscript have the following competing interests: RC has received a consultant fee from Galapagos and is employed by IQVIA outside of the present work. LD has received research grant (paid to her institution) from BMS outside the current manuscript.  She is a member of the steering committee of the Danish Rheumatology Quality Registry (DANBIO, DRQ), which receives public funding from hospital owners and funding from pharmaceutical companies. MLF, EVY, AMB have declared that no competing interests exist.”

Please confirm that this does not alter your adherence to all PLOS ONE policies on sharing data and materials, by including the following statement: "This does not alter our adherence to PLOS ONE policies on sharing data and materials.” (as detailed online in our guide for authors [http://journals.plos.org/plosone/s/competing-interests](https://eur03.safelinks.protection.outlook.com/?url=http%3A%2F%2Fjournals.plos.org%2Fplosone%2Fs%2Fcompeting-interests&data=05|01|enriqueta.vallejo@unibe.ch|a40eb79e005345a92a0208db669c5a96|d400387a212f43eaac7f77aa12d7977e|1|0|638216594270170134|Unknown|TWFpbGZsb3d8eyJWIjoiMC4wLjAwMDAiLCJQIjoiV2luMzIiLCJBTiI6Ik1haWwiLCJXVCI6Mn0%3D|3000|||&sdata=ZSh0eEryQzUdc4xR1KTVBhu%2FWn%2By8BNuZvqp5U6JjLg%3D&reserved=0)).  If there are restrictions on sharing of data and/or materials, please state these. Please note that we cannot proceed with consideration of your article until this information has been declared.

Please include your updated Competing Interests statement in your cover letter; we will change the online submission form on your behalf.

### Answer

We have added the required sentence. Following our updated “Competing interests” section:

Page 2, lines 27 - 32

“RC has received a consultant fee from Galapagos and is employed by IQVIA outside of the present work. LD has received research grant (paid to her institution) from BMS outside the current manuscript. LD is a member of the steering committee of the Danish Rheumatology Quality Registry (DANBIO, DRQ), which receives public funding from the hospital owners and funding from pharmaceutical companies. MLF, EVY, AMB declare no competing interests. This does not alter our adherence to PLOS ONE policies on sharing data and materials.”

## Journal requirement #3

Please provide additional details regarding participant consent. In the ethics statement in the Methods and online submission information, please ensure that you have specified what type you obtained (for instance, written or verbal, and if verbal, how it was documented and witnessed). If your study included minors, state whether you obtained consent from parents or guardians. If the need for consent was waived by the ethics committee, please include this information.

### Answer

Our study does not require patient informed consent, and neither it does the DANBIO Database. This study was approved by the DANBIO (ref. DANBIO-2018-11-30) steering committee and registered at the North Region's inventory (ref. P-2018–182). To support our statement, we amended the “Materials and Methods” section of the manuscript and added two additional references, as indicated below.

Page 7, lines 147 - 159:

“Ethics statement

The study complies with the Declaration of Helsinki. Registry-based research does not require approval from the Danish National Research Ethics Committee. The study was approved by the DANBIO (ref. DANBIO-2018-11-30) steering committee and registered at the North Region's inventory (ref. P-2018–182).

Patient consent for publication

DANBIO is approved as a national quality registry and by the Danish Data Protection Agency, which is based on the Act on Processing of Personal Data and ensures that data security, protection and individual rights, are dealt with correctly. This constitutes the necessary legal requirements, and informed consent is not required per Danish legislation(1). Therefore, informed patient consent is not needed to register or participate in the DANBIO registry.(2)”

## Journal requirement #4

We note that you have stated that you will provide repository information for your data at acceptance. Should your manuscript be accepted for publication, we will hold it until you provide the relevant accession numbers or DOIs necessary to access your data. If you wish to make changes to your Data Availability statement, please describe these changes in your cover letter and we will update your Data Availability statement to reflect the information you provide.

### Answer

Thank you for the information. This has been a misunderstanding. The data belongs to the data holder, DANBIO, and we cannot make it available to third parties. Therefore, data is only available through the data holder (DANBIO) and upon their approval and permission. To clarify that, we added the following text to the “Materials and Methods” section of our manuscript:

Page 8, lines 169 - 174

“Data availability statement

Some data can be shared upon reasonable request to the corresponding author. Data-sharing enquiries will be handled per the data-handling laws from the European General Data Protection Regulation (GDPR). The Danish Data Protection Agency and Statistics Denmark prohibit extraction and sharing of any data making individuals identifiable.”

## Journal requirement #5

Please include a separate caption for each figure in your manuscript.

### Answer

Thank you. We have added a separate caption for each figure at the end of the manuscript, between Author contributions and References (Pages 19 and 20).

## Journal requirement #6

Please include captions for your Supporting Information files at the end of your manuscript, and update any in-text citations to match accordingly. Please see our Supporting Information guidelines for more information: [http://journals.plos.org/plosone/s/supporting-information](https://eur03.safelinks.protection.outlook.com/?url=http%3A%2F%2Fjournals.plos.org%2Fplosone%2Fs%2Fsupporting-information&data=05|01|enriqueta.vallejo@unibe.ch|a40eb79e005345a92a0208db669c5a96|d400387a212f43eaac7f77aa12d7977e|1|0|638216594270170134|Unknown|TWFpbGZsb3d8eyJWIjoiMC4wLjAwMDAiLCJQIjoiV2luMzIiLCJBTiI6Ik1haWwiLCJXVCI6Mn0%3D|3000|||&sdata=S6U4uE4z6KSLmkyLATn%2F9pYDd%2Faz%2BOwMPGKTnYLSodk%3D&reserved=0).

### Answer

Thank you. We have added a separate caption for each Supporting Information file at the end of the manuscript, between Author contributions and References (Pages 20 and 21).

# Reviewer #1

## Reviewer #1 Comment 1

The authors plan to study the prevalence, and incidence, of serious viral infections, VTE and cardiac events in the DANBIO registry comparing JAKis with TNFis treated patients. Their methodology is quite sound and they have done a good job in presenting it. However, and this is important, the reader is unable to judge the representativeness and generalizability of their approach. This is because the Danish health care system approves minimal disease criteria in order to be eligible to receive either a b or tsDMARD. These criteria differ across national health care systems. These European eligibility criteria are typically quite different from country to country and are very different from eligibility criteria in the US. This is reflected by the very much different prevalence of prescribing both b and tsDMARDs in single national health care systems from country to country.

Therefore, the authors must present plans to supply data on:

1. Eligibility to receive both b and tsDMARDs in the Danish system;

2. Percentage of patients (prevalent and incident described separately) who are prescribed these agents with the overall percentage of patients treated in the health care system used as a denominator;

3. Describe how the population studied in this selective population might affect the results they report.

That is, if only patients with more severe disease receive these agents in Denmark, while patients with much less (mean and median) standard disease metrics receive these same drugs in other countries, then these differences are potentially very relevant and affect the generalizability and representativeness of other populations around the world who access these drugs with different baseline disease activity criteria. National health care systems are able to set their own minimal disease criteria based upon a policy of willingness to pay for different disease activity levels.

### Answer

Thank you for the positive feedback on the study methodology and its presentation. We also appreciate the interesting points raised by the reviewer. Below we address each topic individually.

Point 1. Eligibility to receive both b and tsDMARDs in the Danish system;

### Answer

According to the Danish national guidelines, there is an indication of treatment with bDMARDs/ tsDMARDS if:

1. Aktuel og vedvarende, uacceptabel sygdomsaktivitet dokumenteret ved mindst to kliniske kontroller, eller behandlingsmål for remission/LDA ikke er opnået ved højst 3-4 måneder, dokumenteret ved hyppige kontroller (hver 1. til 3. måneder).
2. Ekspertvurderet inflammatorisk aktiv og derved forventet reversibel sygdom.
3. Behandlingserfaring med kombinationsbehandling, primært fortrukne er tripel csDMARD-terapi (MTX og salazopyrin og hydroxychloroquin) i relevant dosering. Ved forekomst af dårlige prognostiske faktorer (se punkt 9.1.3) kan man overveje at gå direkte videre til biologisk behandling efter svigt af methotrexat
4. Indikation for biologisk behandling træffes ved konference med deltagelse af speciallæger i reumatologi

English translation:

1. Actual continuously, unacceptable disease activity in at least two clinical consultations or remission/LDA are not achieved in 3-4 months, documented by frequent consultations (every 1 to 3 months) - actual continuously, unacceptable disease activity in at least two clinical consultations or remission/LDA are not achieved in 3-4 months;

2.	Expert-assessed inflammatory active and thereby expected reversible disease - active, reversible inflammation according to expert;

3.	Treatment experience with combination therapy, primarily preferred is triple csDMARD therapy (MTX and salazopyrin and hydroxychloroquine) in relevant dosages. In the presence of poor prognostic factors (see section 9.1.3), direct progression to biological therapy may be considered after methotrexate failure. - no effect of triple csDMARD treatment or bad prognostic factors present;

4. indication for biological treatment is made in a conference with participation of specialists in rheumatology - the decision is made in a conference with participation of specialists in rheumatology.

Point 2. Percentage of patients (prevalent and incident described separately) who are prescribed these agents with the overall percentage of patients treated in the health care system used as a denominator;

### Answer

### The DANBIO registry was initiated in 2000 and provides nationwide data on the disease course of patients with inflammatory rheumatic diseases treated in routine care.(2) The registry, which is approved as a national quality registry, includes patients with RA, psoriatic arthritis, and ankylosing spondylitis, who are followed longitudinally. The validity of the diagnosis is high.(3) Data are captured electronically from the source (patients and health personnel). By January 1^st^ 2020, DANBIO had since its start included > 35,000 patients with RA. At some point in time, >30,000 of those patients were at some time treated with conventional DMARD, >10,000 of those patients had at some time point received bDMARD and >800 of them a JAKi. More than 22,000 of the DANBIO patients had at least one visit in DANBIO during 2019 with corresponding numbers of >20,000 (csDMARD), >7,500 (bDMARD) and >800 (JAKi). The public health system reimburses drug costs. Although some regional differences have been reported, access to treatment is considered to be better than in most other countries. Baseline data regarding disease activity are considered valid, since the treatment criteria are widely accepted among rheumatologists and patients.

Point 3. Describe how the population studied in this selective population might affect the results they report.

**Answer:**

We agree that studies conducted in DANBIO are representative of the Danish population and not necessarily other populations, especially if they have different population characteristics and healthcare systems. We will provide a complete overview of the patient characteristics within Table 1 of the study manuscript to ensure full transparency of the patient population for comparative purposes.

The reviewer is correct that the healthcare model in Denmark is quite different from that of the US. The Danish model is more comparable to Canada, Italy, or France, where there is a single-payer Universal healthcare system. Thus, patients in Denmark do not pay for bDMARDS or JAKi, but they get it in the hospital for free. Conversely, patients pay for csDMARDs in the Pharmacy (except MTX subcutaneous). As a result, we would not expect to observe substantial socio-economic differences between patients receiving a JAKi compared to earlier, less-expensive options.

Regarding access, JAKis are eligible as a third-line therapy option after csDMARDs and bDMARDs. Thus, patients receiving JAKis within Denmark will have failed or be contraindicated for bDMARDs prior to receiving a JAKi. Clinical practice in Denmark traditionally follows the EULAR guidelines, and therefore, we expect our results to be representative of other countries following EULAR guidelines.

While we will describe the patients in detail – both prevalent and incident users – it is not realistic to describe all patients in the Danish healthcare system as this would be the entire population of Denmark. However, we expect that a detailed description of the JAKis (prevalent and incident) and the bDMARD users will be sufficient to provide an adequate comparison to other countries and databases.

To adapt the protocol, we have now included the following additions to our “Data source” section:

Pages 6 and 7, lines 137 - 145

*“The Danish healthcare model is similar to other countries such as Canada, Italy, or France, where there is a single-payer Universal healthcare system. Thus, patients in Denmark receive bDMARDs or JAKi at no cost as they are provided free of charge within the hospital setting. Regarding access to medication, JAKis are eligible as a third-line therapy option, after csDMARDs and bDMARDs. Thus, patients receiving JAKis within Denmark will have failed or be contraindicated for bDMARDs prior to receiving a JAKi. Finally, clinical practice in Denmark traditionally follows the European Alliance of Associations for Rheumatology (EULAR) guidelines,*(4) *and therefore, we expect our results to be representative to other countries following EULAR guidance.”*

In addition, we have included the following additions to our “Discussion” section:

Pages 18 - 19, lines 475 - 484

*“This protocol has been designed for the DANBIO registry, and thus, a Danish patient population. Denmark has a universal healthcare system and follows the EULAR guidelines for treatment recommendations, and as such, it may have some inherent differences from other countries. For example, we may expect fewer socio-economic differences between our patient groups and will provide comparisons between our cohort and other published studies. However, one of the strengths of the study design is that the prevalent-new user design with propensity score matching addresses circumstances in which the study and reference drugs are not prescribed under the same criteria. While we would not expect substantially different results in different patient settings, it is possible that finding optimal bDMARD matches for JAK inhibitor patients may be more challenging in some populations.”*

# Reviewer #2

## Reviewer #2 General comment

Thank you for giving me the opportunity to review this article. I have carefully read the article. Although there are important safety data from individual RTCs as well as from pooled analyses on RCTs, real-world data comparing safety of JAKis and TNFis are currently limited. Therefore, the aim of the study serves to provide valuable safety knowledge on JAKis treatment of RA patients.

The article deals with the study design and methodology of the planned prospective cohort study, and overall, the study design has been elaborately made to avoid confounding. However, some part of the description lacks detailed explanation. I suggest that the followings be explained in more detail.

### Answer

We appreciate the reviewer's careful reading and review of our manuscript.

## Reviewer #2 Comment 1

Different abbreviations (DNPR and NPR) were used for the same meaning in the “DATA SOURCE“ section.

### Answer

Thank you for this comment. We understand that both the Danish National Patient Registry (DNPR) and the Danish National Prescription Registry (NPR) have very similar names and, hence, very similar acronyms. Therefore, we have removed the acronyms and used the full wording for both registries. This applies to the Data source section (within the Materials and methods section) and Discussion section.

## Reviewer #2 Comment 2

For Figure 1. incorporate the difference between MACE/VTE cohort and viral infection cohort.

### Answer

We appreciate this comment, and we have split Figure 1 into two Figures, one per cohort (see below), to indicate better the different exclusion criteria that lead to the MACE and VTE cohort (Figure 1) and the severe viral infection cohort (Figure 2).

Thus, to adapt the protocol, we have now split Figure 1 into two figures:

*“****Figure 1. Base cohorts for MACE and VTE outcomes, inclusion and exclusion criteria, exposure sets, and study cohorts.*** *Abbreviations: RA Rheumatoid Arthritis; JAKi Janus Kinase inhibitor; TNF-αi Tumor Necrosis Factor α inhibitor; DANBIO Danish Rheumatologic database; MACE major adverse cardiovascular events; VTE venous thromboembolism. TCPS time-conditional propensity score; T_0_ will be defined as the study cohort entry date for JAKi users (i.e., first start of JAKi treatment) and the corresponding matching date for TNF-αi users.”*

*“****Figure 2. Base cohorts for the outcome of Serious viral infection, inclusion and exclusion criteria, exposure sets, and study cohorts.*** *Abbreviations: RA Rheumatoid Arthritis; JAKi Janus Kinase inhibitor; TNF-αi Tumor Necrosis Factor α inhibitor; HIV Human Immunodeficiency Virus; DANBIO Danish Rheumatologic database. TCPS time-conditional propensity score; T_0_ will be defined as the study cohort entry date for JAKi users (i.e., first start of JAKi treatment) and the corresponding matching date for TNF-αi users.”*

## Reviewer #2 Comment 3

Figure 1 should come after the paragraph “base cohort”.

### Answer

Following this comment, we have removed the note “*[Figure 1 should be placed here]*” (Page 9), and we keep to the discretion of the editors to place Figures 1 and 2 with base cohorts where they see fit.

## Reviewer #2 Comments 4 and 5

In Figure 2, only prevalent new user of JAKi and his or her potential controls are depicted. Please make it include the incident new users.

In Figure 2, describing why patients 3 and 4 are not potential controls will help readers to understand the study cohort selection process.

### Answer

We appreciate the comments on previous Figure 2 (which in the amended protocol manuscript will become Figure 3) and have modified it (see below) to improve clarity. We indicated in the figure the potential TNF-αi controls for JAKi users in prevalent and new users (Figure 3a and Figure 3b, respectively). Additionally, we described in the figure why patients were or were not potential controls to better depict the selection of potential controls for the exposure sets. Finally, we included two examples of different TNF-αis, infliximab and etanercept, to Illustrate the eligibility criteria (ii).

Thus, we have revised the previous Figure 2 (new Figure 3) to adapt the protocol manuscript.

*“****Figure 3.*** ***Graphical description of the time-dependent exposure set for Janus Kinase inhibitors (JAKis) users with previous use of Tumor Necrosis Factor inhibitors (TNF-αis) (a) and with no previous use of TNF-αis (b).*** *In* ***Figure 3a****,* *we depict an example of potential controls (patients A2, A3, A4, A5) to be included in the exposure set for a JAKi user with previous TNF-αi use (patient A1), and three examples (patients A6, A7, and A8) who are not eligible to be controls for that JAKi user based on: (i) calendar time (TNF-αi use within ± 90 days from T_0_), (ii) type of TNF-αi at T_0_ (not applicable for JAKis users with no previous use of TNF-αi), (iii) time since the start of the patient’s first TNF-αi drug within a difference of ± 90 calendar days (i.e., look back period from study cohort entry date up to base cohort entry), and (iv) duration (days) of the most recent TNF-αi treatment course, starting looking from T_0_ and continue backwards in time until a period free of treatment > 90 days. Additionally, in* ***Figure 3b****, potential controls (patients B3 and B4) to be included in the exposure set for a JAKi user with no previous TNF-αi use (patient B1), and one example (patient B2) who is not eligible to be a control for that JAKi user based on the requirements above (i), (iii), and (iv). While infliximab and etanercept were used to exemplify patients using different TNF-α drugs, the list of TNF-αis used in the study is not restricted to these two drugs.* *Abbreviations: NA not applicable; JAKi Janus Kinase inhibitor; TNF-αi tumor necrosis factor α inhibitor; T_0_ will be defined as the study cohort entry date for JAK inhibitor users and the corresponding matching date for TNF-αi users.”*

## Reviewer #2 Comment 6

In “Base Cohort” section, the sentence “Note that the base cohort entry may be previous to the study period for TNFi users” will be better placed in “Exposure set” section since such discrepancy occurs when matching a TNFi user to a prevalent new user of a JAKi. Figure 2 will help understanding, which is also being placed in “Exposure set” section.

### Answer

As suggested by the reviewer, we have moved the mentioned sentence from the Base cohort section to the Exposure sets section.

Page 10, lines 247 - 248

“Note that the base cohort entry may be previous to the study period for TNF-αi users.”

## Reviewer #2 Comment 7

There are four levels of study populations: study population, base cohort, exposure sets, and study cohort, which makes the readers confusing. Please add explanations for the purpose of this multi-level populations “upfront” and specify the role of each population.

### Answer

Thank you for this comment. Aiming to clarify these concepts and their purpose, first, we incorporated “study population” into “base cohort” as the difference between both was minimal. This applies to the “Study population” section (within the Materials and methods section) and “Base cohort” section.

Then, we added the following text to the “Base cohort” section.

Pages 8 and 9, lines 194 - 207

“A graphical representation of base cohorts for MACE/VTE outcomes and Serious viral infection, depicting inclusion and exclusion criteria, is shown in **Figure 1** and **Figure 2**, respectively. Starting from patients in the DANBIO registry database, we may define subsequent key population steps in this study: base cohorts, exposure sets, and study cohorts. These are explained in detail later in the protocol, here in brief. As illustrated in **Figure 1** and **Figure 2**, a base cohort will be obtained after applying the inclusion and exclusion criteria. This will include JAKis and TNF-αi initiators (i.e., potential controls) starting the drug treatment within the study period. From the base cohort, JAKis users will be identified as members of the JAKi group and subsequently matched with selected TNF-αi users within their exposure set. For that, the exposure set for each JAKi user will include a pool of TNF-αi users from the base cohort who are eligible to be their controls (i.e., due to similar exposure until the time of JAKi start). Notably, the exposure sets are the distinction between prevalent and incident users of JAKs. Lastly, the matched JAKi users and TNF-αi users with similar treatment history at the moment the JAKi start constitute the study cohorts.”

## Reviewer #2 Comment 8

Do authors distinguish switching between different TNFis? Authors did not specify whether they will censor at switching from one TNFi to a different TNFi, which makes me assume that the authors will treat TNFis as a class. However, in Figure 2, the exposure set condition includes types of TNFi.

### Answer

The reviewer highlights a good point. While we consider the type of TNF-αi during the selection of the exposure set for each JAKi user, we treat every TNF-αi as a class during follow-up, with no particular action linked to the switch of TNF-αi type. To clarify this in the manuscript, we added a sentence in the “Follow-up and censoring section” (within the Materials and methods section).

Page 12, line 298

*“During follow-up, the TNF-αis will be treated as a class*.”

## Reviewer #2 Comment 9

Please present the PPV of each outcome definition for the DNPR. If there is no PPV made for ICD10 used with DNPR, describe possibility of the misclassification bias.

### Answer

Thank you for capturing attention on this point. The validity of cardiovascular diagnoses in the Danish National Patient Registry (DNPR) was previously investigated and is overall high and sufficient for use in research.(5) For example, PPV for first-time myocardial infarction and thromboembolism exceed 90%. Regarding serious viral infection, the PPV of serious viral infection diagnoses in the DNPR is unknown. Nevertheless, previous studies have found high diagnostic accuracy of HZ with PPV ranging from 72% to 100 % worldwide.(6–10) Moreover, the PPV for first-time HZ-specific prescriptions (i.e., acyclovir, valacyclovir, and famciclovir) in Denmark was found to be 87%.(11)

Despite the overall completeness and validity of diagnoses in the Danish National Patient Registry,(12) the potential for bias due to the misclassification of outcomes should not be ruled out of this study. This is an intrinsic limitation, given that the data is collected for non-research purposes. Therefore, to clarify this in the manuscript, we added a sentence in the “Discussion” section:

Page 19, lines 479 - 482

*“Finally,* despite the overall completeness and validity of outcome diagnoses in DNPR,(12) the potential for bias due to misclassification of outcomes should not be completely ruled out. This is an intrinsic limitation of RWD-based studies, as data is often collected for non-research purposes.”

## Reviewer #2 Comment 10

Follow-up and censoring as a result of a change in the exposure status, which will be defined as (i) treatment discontinuation (defined by recorded treatment discontinuation or a gap of >90 days, in which case we assume stop at start/stop of the gap) please rephrase the underline part since it is hard to follow.

### Answer

We appreciate the reviewer's comment and we have rephrased the related text.

Page 12, lines 290 - 297

“We will follow all patients in the study cohort from the study cohort entry date until the earliest of the following: occurrence of first ICD-10 code for an outcome event, end of study period, or censoring as a result of a change in the exposure status. Change in exposure status will be defined as (i) treatment discontinuation (defined by the earliest of the following: a recorded treatment discontinuation date, or at the beginning of a treatment gap of >90 days) or (ii) switching from a JAKi to a non-JAKi drug, or (iii) switching from a TNF-αi to a non-TNF-αi drug, or (iv) death. We will accept a permissible gap of up to 90 days between courses of the same exposure treatment.”

## Reviewer #2 Comment 11

Ascertainment periods for individual covariates are different. Briefly explain on which criteria each of the time period was set.

### Answer

Addressing the reviewer’s comment, we included the below sentence in the Covariates section within the “Materials and methods” section.

Page 13, lines 317 - 318

“Ascertainment periods for covariates in Table S5 and Table S6 were chosen based on clinical and methodological rationale.”

## Reviewer #2 Comment 12

It is not clear whether the matching is fixed ratio (1:4) or variable ratio. Please specify.

### Answer

Thank you for the comment, and we apologize for not specifying it. We will use variable ratio matching, and thus, each JAKi user will be matched with up to 4 TNF-αi users.

To clarify that, we modified the following sentence in the “Time-conditional propensity score (TCPS) matching section” of the manuscript:

Page 14, lines 342-346

*“We will match JAKi users to TNF-αi users within exposure sets on nearest TCPS and on a variable ratio (i.e., one JAKi user will be matched with up to four TNF-αi controls), without replacement and in chronological order.”*

***ADDITIONAL COMMENTS FROM THE CO-AUTHORS & FORMATTING AMENDMENTS***

***Comment #1***

We removed references 17 and 29 from the manuscript, which were identical to reference 11 (please see below).

Ytterberg SR, Bhatt DL, Mikuls TR, Koch GG, Fleischmann R, Rivas JL, et al. Cardiovascular and Cancer Risk with Tofacitinib in Rheumatoid Arthritis. N Engl J Med. 2022 Jan 27;386(4):316–26.

***Comment #2***

To improve clarity, we rephrased the following sentence in the “Data Source” section.

Page 6, lines 116 - 118

*“The unique civil registration number (CPR number) from the Danish Civil Registration System (13) enables linkage between these national data sources on an individual-based level.”*

**References:**

1.	Thygesen LC, Daasnes C, Thaulow I, Brønnum-Hansen H. Introduction to Danish (nationwide) registers on health and social issues: structure, access, legislation, and archiving. Scand J Public Health. 2011 Jul;39(7 Suppl):12–6.

2.	Ibfelt EH, Jensen D, Hetland ML. The Danish nationwide clinical register for patients with rheumatoid arthritis: DANBIO. CLEP. 2016 Oct;Volume 8:737–42.

3.	Ibfelt EH, Sørensen J, Jensen DV, Dreyer L, Schiøttz-Christensen B, Thygesen PH, et al. Validity and completeness of rheumatoid arthritis diagnoses in the nationwide DANBIO clinical register and the Danish National Patient Registry. CLEP. 2017 Nov;Volume 9:627–32.

4.	Smolen JS, Landewé RBM, Bergstra SA, Kerschbaumer A, Sepriano A, Aletaha D, et al. EULAR recommendations for the management of rheumatoid arthritis with synthetic and biological disease-modifying antirheumatic drugs: 2022 update. Annals of the Rheumatic Diseases. 2023 Jan 1;82(1):3–18.

5.	Sundbøll J, Adelborg K, Munch T, Frøslev T, Sørensen HT, Bøtker HE, et al. Positive predictive value of cardiovascular diagnoses in the Danish National Patient Registry: a validation study. BMJ Open. 2016 Nov;6(11):e012832.

6.	Oxman MN, Levin MJ, Johnson GR, Schmader KE, Straus SE, Gelb LD, et al. A Vaccine to Prevent Herpes Zoster and Postherpetic Neuralgia in Older Adults. N Engl J Med. 2005 Jun 2;352(22):2271–84.

7.	Rübben A, Baron JM, Grussendorf-Conen EI. Routine detection of herpes simplex virus and varicella zoster virus by polymerase chain reaction reveals that initial herpes zoster is frequently misdiagnosed as herpes simplex. Br J Dermatol. 1997 Aug;137(2):259–61.

8.	Scott FT, Johnson RW, Leedham-Green M, Davies E, Edmunds WJ, Breuer J. The burden of Herpes Zoster: a prospective population based study. Vaccine. 2006 Feb 27;24(9):1308–14.

9.	Weinmann S, Chun C, Schmid DS, Roberts M, Vandermeer M, Riedlinger K, et al. Incidence and clinical characteristics of herpes zoster among children in the varicella vaccine era, 2005-2009. J Infect Dis. 2013 Dec 1;208(11):1859–68.

10.	Rimland D, Moanna A. Increasing incidence of herpes zoster among Veterans. Clin Infect Dis. 2010 Apr 1;50(7):1000–5.

11.	Schmidt SAJ, Vestergaard M, Baggesen LM, Pedersen L, Schønheyder HC, Sørensen HT. Prevaccination epidemiology of herpes zoster in Denmark: Quantification of occurrence and risk factors. Vaccine. 2017 Oct 9;35(42):5589–96.

12.	Schmidt M, Schmidt SAJ, Sandegaard JL, Ehrenstein V, Pedersen L, Sørensen HT. The Danish National Patient Registry: a review of content, data quality, and research potential. Clin Epidemiol. 2015;7:449–90.

13.	Pedersen CB. The Danish Civil Registration System. Scand J Public Health. 2011 Jul;39(7_suppl):22–5.
